# Physiopathological aspects of the subclinical alterations of thyroid function associated with Acute Coronary Syndromes

**Published:** 2013-12-25

**Authors:** CS Stamate, AM Andronescu, AC Nechita, C Delcea, EM Mihu, MM Vintila

**Affiliations:** * 1st Internal Medicine and Cardiology Department, “Sfantul Pantelimon” Clinical Emergency Hospital Bucharest; ** „Carol Davila” University of Medicine and Pharmacy Bucharest

**Keywords:** subclinical thyroid dysfunction, acute coronary syndrome, cardiac risk factor

## Abstract

Abstract

The subclinical modification of thyroid function represents an important risk factor for the development of acute coronary syndromes, neglected up to this day. Knowledge of the physiopathological processes implicated in the alteration of thyroid function that induces cardiovascular dysfunction is a necessity for the understanding of the phenomena and for the finding of the adequate therapeutic solutions.

While recognizing the thyroid dysfunction as a modifiable risk factor for the acute coronary syndrome, we encountered a new challenge for the clinical research regarding its implications.

The ability to manage the altered thyroid homeostasis may represent a new stage of prevention at a population level for the reduction of the cardiac risk, a stage which implies a risk factor that may remain clinically mute for a long period of time if left undiagnosed, however influencing the development of the acute coronary syndromes.

## Introduction

In spite of the important scientific progress which was made recently, the cardiovascular pathology is still the leading cause of mortality in Romania, our country occupying, unfortunately, the first place in Europe regarding this matter.

Thus, the concern regarding primary as well as secondary prevention of cardiovascular pathology must have important implications for the entire medical body. The efforts made to correct modifiable risk factors such as diabetes mellitus, dyslipidemia, arterial hypertension, sedentarism, obesity, smoking are well known and with high impact on the general population.

The relationship between thyroid hormonal secretion and cardiac activity is reputable in the clinical setting mentioned above. Lately, numerous studies have focused on the impact of subclinical forms of thyroid dysfunction upon the development of cardiovascular disease, especially of acute coronary syndromes [**1**-**3**].

**Thyroid hormones – Mechanism of action**

As it is well known, the thyroid gland is under the hormonal control of the hypothalamo-hypophyseal system, which, by feed-back mechanism, influences the circulating levels of thyroid hormones.

The thyroid gland is not only controlled by this mechanism, but also by the following:

−the intrinsic mechanism based on the relationship between the intrathyroid organic iodine and the rate of hormonal secretion

−the influence of different hormonal or nutritional factors as well as factors playing a role in different physiopathological disorders, the so-called extrathyroid factors

−anti-TSH receptor autoantibodies with intrinsic, independent dynamic

−hypophyseal and peripheral deiodinases that modulate the thyroxin and triiodothyronine effects [**4**,**5**].

Responding to TSH secretion, the thyroid gland synthesizes thyroxin (T4) and triiodothyronine (T3). Secreted levels of triiodothyronine represent less than 5% of the entire thyroid secretion. The vast majority of active compounds of triiodothyronine are a result of the conversion of thyroxin into triiodothyronine by a deiodination process.

The role of triiodothyronine is then exerted on isoform type α receptors, either α1 or α2 and type β receptors, either type β1, β2 or β3 [**4**,**6**,**7**]. Regarding their cardiac distribution, these receptors are located both on atrial cells as well as on the ventricular ones [**8**]. By binding to these receptors, the thyroid hormones influence the accelerated myosin synthesis, the sarcoplasmic reticulum activity, the movement through the ionic Ca and K channels, the response of the adrenergic receptors, the transmembrane ion gradients, the levels of ATP, as well as those of the atrial natriuretic peptide [**9**-**11**].

**The effects of thyroid hormones on cardiovascular function**

The effects of thyroid hormones must be categorized as genomic and non-genomic, that structurally and functionally influence the cardiovascular proteins [**12**,**22**]. Acting on the α receptors, triiodothyronine plays a role in the process of increasing the myocardial contractility and enhancing the production of myosin. Acting in the β receptors, it influences the diastolic processes as well as the left ventricular relaxation [**9**].

**Table 1:** Genomic effect of the regulating role of thyroid hormones

−	+
β miosin synthesis	α miosin synthesis
Ionic gradient Na/Ca - α 1 thyroid receptors	Sarcoplasmic reticulum ATP-ase
Type V and VI Adenyl Cyclase	Atrial natriuretic peptide
Gi Nucleotide – Guanine Binding	Gs Nucleotide – Guanine Binding

The main mechanism is that of reducing the high levels of citosolic calcium during the systolic function to lower, diastolic levels [**13**]. This effect is best outlined when associated with the circadian catecholamine secretion rhythm, when the excess thyroid hormone levels produce the “sleep tachycardia” thus proving different mechanisms of action.

Regarding its function on a vascular level, the essential role of triiodothyronine is that of participating in the maintenance and renewal of endothelial integrity, as well as on peripheral arterial resistance, at the same time modulating the arterial response to the activation of the rennin-angiotensin-aldosterone mechanism [**14**-**16**].

This hormone also controls the macrophage reaction to the deposition of lipids in the vascular wall [**17**].

Apart from these direct effects, the thyroid hormones play an important role in the development of cardiovascular pathology by other mechanisms as well, such as:

 −modifying the renal absorption of sodium

−influencing the erythropoietin synthesis

−influencing the coagulation process by controlling the levels of activated factor VII as well as the ratio of activated factor VII and anti-activated factor VII antigen.

**Physiopathological aspects involved in the development of acute coronary syndromes in patients with thyroid endocrine dysfunction**

It should be noted that recent studies address more often the relationship between the acute coronary syndrome and the subclinical thyroid dysfunction, expressed only by the alteration of the levels of one circulating hormone, especially TSH, even if the remaining thyroid hormone levels are normal [**19**-**22**]. From a pathophysiological point of view, hypothyroidism or hyperthyroidism, generally have opposite effects. Hyperthyroidism reduces the systemic vascular resistance, the diastolic blood pressure, the afterload and increases the vessel wall thickness and stiffness, the chronotropism and inotropism, the cardiac output per minute and the total blood volume. Hyperthyroidism is responsible for the occurrence of tachycardia and in particular atrial tachyarrhythmias and it reduces heart rate variability. At the same time, however, it increases oxygen consumption in the myocardial fibers.

**Table 2:** Main cardiovascular changes

Parameter	Hypothyroidism	Hyperthyroidism
Arterial wall thickness stiffness	↑	↑
Cardiac chronotropy and inotropy	↓	↑
Afterload	↑	↓
Cardiac output basal	↓	↑
Diastolic blood pressure	↑	↓
Blood volume	↓	↑
Systemic volume resistance	↑	↓

Hypothyroidism reduces the cardiac output per minute, the blood volume, the chronotropism and inotropism, and it increases the systemic vascular resistance, the diastolic blood pressure, the vascular wall thickness and stiffness as well the afterload. The increase of the peripheral resistance mainly induces a systolic dysfunction of the left ventricle, abnormal relaxation, without the modification of the heart rate.

Changes in the arterial wall elasticity are involved in the progression of atherosclerotic processes.

By affecting the vascular endothelial function, alterations occur in blood flow, with the nitric oxide playing an important role in this process.

Hypothyroidism decreases glomerular filtration rate, which influences circulating cholesterol levels and at the same time favors the development of type II diabetes complications [**23**-**28**].

**Sub-clinical forms of thyroid dysfunction – a hidden enemy?**

The scientific support of clinical studies based on a small and medium number of patients (up to 850 patients for each study) up to this moment, seems to offer credible arguments for a serious assessment of the relation between the acute coronary syndrome and the subclinical forms of thyroid dysfunction. While in the current medical practice we are accustomed to the clinically manifest forms, the subclinical forms are, many times, undiagnosed, without previous arguments for them to be considered in the differential or positive diagnosis.

The subclinical forms are referring to the alteration of the circulant level of only one of the main active hormones, modification which does not surpass a medium value. The endocrine dysfunction of triiodothyronine/ thyroxine ratio mainly affects the left ventricle. The patients with low triiodothyronine levels have a lower survival rate after a major coronary event or after heart failure symptoms, as a complication of an acute coronary syndrome. The decrease of the circulating triiodothyronine level with normal TSH is associated with an important decrease of HDL cholesterol levels, the increase of neutrophil count and decrease of lymphocyte count [**29**-**38**].

Subclinical hypothyroidism is defined as an increase in the circulating levels of TSH, associated with a normal level of thyroxin. Some studies report the presence of subclinical hypothyroidism in 10-15% of the patients with acute coronary syndromes. Prevalence is higher in women than in men, with an evolution of the acute coronary syndrome severity correlated with age. Also, subclinical hypothyroidism is associated with the presence of dyslipidemia and premature development of atheromatosis [**18**,**39**-**45**].

The development of acute coronary syndromes in patients with subclinical hypothyroidism is induced not only by dyslipidemia but also by the influence of this hormonal imbalance on the coagulation system, on vasodilatation, on the parasympathetic activity and homocysteine metabolism [**46**-**52**].

Currently, the treatment of subclinical forms still remains poorly understood. Studies showed that attempts to correct circulating thyroid hormone levels had a positive effect in terms of the evolution of heart disease, in patients treated conservatively, but also in interventionally treated patients. Diagnosis of subclinical forms cannot be made unless specifically sought by laboratory measurements.

## Discussion

The difficulty of diagnosing the subclinical forms of thyroid dysfunction, in the absence of clinical manifestations, implies medical thinking in this direction to be determined only by consequent specific laboratory investigations.

In clinical practice, the tendency to measure only TSH and T3/ T4 levels, may sometimes lead to the situation when even in the presence of these laboratory findings, certain thyroid dysfunctions are undiagnosed.

The presence of thyroid dysfunction significantly influences the emergence and development of acute coronary syndromes.

The control of thyroid dysfunction appears to have a favorable effect regarding cardiac pathology development, regardless of the level of prevention. In this setting, the determination of the circulating levels of TSH, triiodothyronine, thyroxine and T3/T4 ratio should be a routine determination, especially in patients with cardiac risk. This way, an important modifiable risk factor would be highlighted, which would otherwise remain "silent" in terms of clinical manifestations for a long period of time.

## References

[1] Ertugrul O, Ahmet U (2011). Prevalence of Subclinical Hypothyroidism among Patients with Acute Myocardial Infarction.. ISRN Endocrinol..

[2] Hak AE, Pols HAP (2000). Subclinical hypothyroidism is an independent risk factor for atherosclerosis and myocardial infarction in elderly women: the rotterdam study.. Ann Intern Med..

[3] Ochs N, Auer R (2008). Meta-analysis: subclinical thyroid dysfunction and the risk for coronary heart disease and mortality.. Ann Intern Med.

[4] Karmisholt J, Andersen S, Laurberg P (2008). Variation in thyroid function tests in patients with stable untreated subclinical hypothyroidism.. Thyroid..

[5] Yen PM (2001). Physiological and molecular basis of thyroid hormone action.. Physiol Rev..

[6] Pavlou HN, Kliridis PA (2002). Euthyroid sick syndrome in acute ischemic syndromes.. Angiology..

[7] Lazar MA (1993). Thyroid hormone receptors: multiple forms, multiple possibilities.. Endocr Rev..

[3] Lazar MA, Chin WW (1990). Nuclear thyroid hormone receptors.. J Clin Invest..

[9] Davis PJ, Shih A (2000). Thyroxine promotes association of mitogen activated protein kinase and nuclear thyroid hormone receptor (TR) and causes serine phosphorylation of TR. J Biol Chem..

[10]  Lazar MA (2003). Thyroid hormone action: a binding contract.. J Clin Invest..

[11] Ojamaa K, Petrie JF, Balkman C (1994). Posttranscriptional modification of myosin heavy-chain gene expression in the hypertrophied rat myocardium.. Proc Natl Acad Sci USA..

[12] Cini G, Carpi A (2009). Thyroid hormones and the cardiovascular system: pathophysiology and interventions.. Biomed Pharmacother..

[13] Frank KF, Bolck B, Erdmann E (2003). Sarcoplasmic reticulum Ca2ρ- ATPase modulates cardiac contraction and relaxation.. Cardiovasc Res..

[14] Vargas F, Moreno JM (2006). Vascular and renal function in experimental thyroid disorders.. Eur J Endocrinol..

[15] Fukuyama K, Ichiki T, Takeda K (2003). Downregulation of vascular angiotensin II type 1 receptor by thyroid hormone.. Hypertension..

[16] Fommei E, Lervasi G (2002). The role of thyroid hormone in blood pressure homeostasis: evidence from short-term hypothyroidism in humans.. J Clin Endocrinol Metab..

[17] Klein I, Ojamaa K (2001). Thyroid hormone and the cardiovascular system.. N Engl J Med..

[18] Cappola AR, Ladenson PW (2003). Hypothyroidism and atherosclerosis.. J Clin Endocrinol Metab..

[19] Kahaly GJ, Dillmann WN (2005). Thyroid hormone action in the heart.. Endocr Rev..

[20] Tieche M, Lupi GA, Gutweller F (1981). Borderline low thyroid function and thyroid autoimmunity. Risk factors for coronary heart disease?. Br Heart J..

[21] Basset JH, Harvey CB,  Williams GR (2003). Mechanisms of thyroid hormone receptor-specific nuclear and extra nuclear actions.. Mol Cell Endocrinol..

[22] Hiroi Y, Kim HH, Ying H (2006). Rapid nongenomic actions of thyroid hormone.. Proc Natl Acad Sci USA..

[23] Parle JV, Franklyn JA, Cross KW (1992). Circulating lipids and minor abnormalities of thyroid function.. Clin Endocrinol..

[24] Sawin CT, Geller A, Wolf PA (1994). Low serum thyrotropin concentrations as a risk factor for atrial fibrillation in older persons.. N Engl J Med..

[25] Toruner F, Altinova AE, Karakoc A (2008). Risk factors for cardiovascular disease in patients with subclinical hypothyroidism.. Adv Ther..

[26] Bastenie PA, Banhaelst L, Bonnyns M (1971). Preclinical hypothyroidism: a risk factor for coronary heart-disease.. Lancet..

[27] Singh S, Duggal J, Molnar J (2008). Impact of subclinical thyroid disorders on coronary heart disease, cardiovascular and all-cause mortality: a metaanalysis.. International Journal of Cardiology..

[28] Biondi B, Cooper DS (2008). The clinical significance of subclinical thyroid dysfunction.. Endocrine Reviews..

[29] Efstathiadou Z, Bitsis S, Milionis HJ (2001). Lipid profile in subclinical hypothyroidism: is L-thyroxine substitution beneficial?.. European Journal of Endocrinology..

[30] Kahaly GJ (2000). Cardiovascular and atherogenic aspects of subclinical hypothyroidism.. Thyroid..

[31] Rodondi N, Aujesky  D, Vittinghoff E (2006). Subclinical hypothyroidism and the risk of coronary heart disease: a meta-analysis.. American Journal of Medicine..

[32] Cappola AR, Fried LP, Arnold AM (2006). Thyroid status, cardiovascular risk, and mortality in older adults.. JAMA..

[33] Chopra IJ, Chopra U, Smith SR (1975). Reciprocal changes in serum concentrations of 3,3’, 5’-triiodothyronine (reverse T3) and 3,5,3’-triiodothyronine(3)T in systemic illnesses.. J Clin Endocrinol Metab..

[34] Wiersinga WM, Lie KI, Touber JL (1981). Thyroid hormones in acute myocardial infarction.. Clin Endocrinol..

[35] Killip T, Kimball JT (1967). Treatment of myocardial infarction in a coronary care unit.. Am J Cardiol..

[36] Kristensen BO, Weeke J (1977). Propranolol-induced increments in total and free serum thyroxine in patients with essential hypertension.. Clin Pharmacol Therap..

[37] Pedersen F, Perrild  H, Rasmussen SL (1984). Low T3- syndrome in acute myocardial infarction-relationship to beta-adrenergic blockade and clinical course.. Eur J Clin Pharmacol..

[38] Kahaly GJ (2000). Cardiovascular and atherogenic aspects of subclinical hypothyroidism.. Thyroid..

[39] Rodondi N, Newman AB, Vittinghoff E (2005). Subclinical Hypothyroidism and the Risk of Heart Failure, Other Cardiovascular Events, and Death.. Arch Intern Med..

[40] Surks MI, Ortiz E, Daniels GH (2004). Subclinical thyroid disease: scientific review and guidelines for diagnosis and management.. JAMA..

[41] Auer J, Beret R, Weber T (2003). Thyroid function is associated with presence and severity of coronary atherosclerosis.. Clin Cardiol..

[42] Canaris GJ, Manowitz NR, Mayor G (2000). The Colorado thyroid disease prevalence study.. Arch Intern Med..

[43] Abohashem-Aly AA, Meng X, Li J (2011). DITPA, a thyroid hormone analog, reduces infarct size and attenuates the inflammatory response following myocardial ischemia.. J Surg Res..

[44] Ross DS (2001). Serum thyroid-stimulating hormone measurement for assessment of thyroid function and disease.. Endocrinol Metab Clin North Am..

[45] Westergreen U, Burger A, Levin K (1977). Divergent changes of serum 3,5,3’-triiodothyronine and 3,3 ’,5 ’-triiodothyronine in patients with acute myocardial infarction.. Acta Med Scand..

[46] Hamilton MA, Stevenson LW, Luu M (1990). Altered thyroid hormone metabolism in advanced heart failure.. J Am Coll Cardiol..

[47] Wrutniak-Cabello C, Casas F, Cabello G (2001). Thyroid hormone action in mitochondria.. J Mol Endocrinol..

[48] Shi W, Wymore R, Yu H (1999). Distribution and prevalence of hyperpolarization- activated cation channel (HCN) mRNA expression in cardiac tissues.. Circ Res..

[49] Kiss E, Jakab G, Kranias EG (1994). Thyroid hormone-induced alterations in phospholamban protein expression: regulatory effects on sarcoplasmic reticulum Ca2ρ transport and myocardial relaxation.. Circ Res..

[50] Koss KL, Kranias EG (1996). Phospholamban: a prominent regulator of myocardial contractility.. Circ Res..

[51] Masunaga R, Nagasaka R, Nakai A (1997). Alteration of platelet aggregation in patients with thyroid disorders.. Metabolism..

[52] Christ-Crain M, Meier C, Guglielmetti M (2003). Elevated C-reactive protein and homocysteine values: cardiovascular risk factors in hypothyroidism? a cross-sectional and a doubleblind, placebo-controlled trial.. Atherosclerosis..

